# Lessons learned from the impact of Covid‐19 on the work of disability support organisations that support employers of social care personal assistants in England

**DOI:** 10.1111/hsc.14098

**Published:** 2022-11-08

**Authors:** Monica Leverton, Kritika Samsi, John Woolham, Jill Manthorpe

**Affiliations:** ^1^ NIHR Policy Research Unit in Health & Social Care Workforce, Policy Institute King's College London London UK

**Keywords:** Covid‐19, direct payments, personal assistants, social care, social care workforce, support services

## Abstract

Social care Personal Assistants (PAs) are directly employed by individuals to assist with activities of daily living such as help or support with personal care, shopping, household tasks and community participation. This option is encouraged by UK public funding. In England, disabled people's support organisations initially offered assistance with such arrangements, although numbers doing this have declined. The Covid‐19 pandemic provided the opportunity to ask those remaining organisations providing support for PA employers about their activities during this time and the questions being posed to them by PA employers. This paper reports data from 15 interviews undertaken March–July 2021 with disability support organisation representatives. We identified one overarching theme ‘Working to prevent and challenge marginalisation of PA employers’, with three related subthemes: (1) Advocating for the voice of a forgotten group; (2) Needing to be proactive and (3) Adapting to new tasks and ways of working. Participant accounts focused on representing the needs of disabled people to the authorities and providing concise, timely and accurate information to PA employers, particularly around the use of public funds during Covid‐19. Remote working amplified the digital‐divide, resulting in these organisations working hard to ensure PA employers received important information about their support options. Befriending services and Covid‐hubs were established by some organisations to reduce isolation and risks of poor mental health amongst PA employers. Many of the challenges facing PA employers existed pre‐pandemic but were perceived to have been heightened during it, reflecting the value of and need for the work of these local support organisations. Our findings suggest areas where effective contingency planning drawn from closer collaboration between disability support organisations and central and local government might usefully be focussed. The potential for specific services or organisations to be commissioned to provide such support is discussed.

**What is known about this subject**

- The Covid‐19 pandemic created multiple challenges for people with care and support needs with increased risks of physical and mental health deterioration
- Disability support organisations can assist people employing Personal Assistants with recruitment, training, direct payments management and administrative responsibilities
- Little is known about staff activity in disability support organisations during Covid‐19

**What this paper adds**

- This study identifies the changing staff activities in disability support organisations during Covid‐19, including new or increased befriending services and mental well‐being checks
- Participants felt they were attempting to compensate for the slow, over‐complicated and not sufficiently specific information being provided by some local authorities to employers of Personal Assistants
- Participants' work involved advocating for the needs of employers of Personal Assistants, driven by perceptions that the pandemic elucidated and heightened existing challenges facing people with care and support needs

## 1 INTRODUCTION

There is increasing interest internationally in the potential for individualised funding to support the well‐being of people needing care and support (Fleming et al., 2019). In England, this is aligned with the personalisation agenda and independent living movement (ILM), where choice and control are at the forefront of policy statements to enable independent living for people with care and support needs. The option of ‘cash for care’ through personal budgets (i.e. a direct payment [DP]) was promoted to implement these policy goals, aiming to transform social care in England by empowering the budget holder or care user to be in control of designing, resourcing and budgeting of their support (Miller et al., 2021). Employing Personal Assistants (PAs) was advocated as providing a more personalised relationship between the employer and their support staff. PAs are employed directly by care users (although some are self‐employed and so responsible for their own tax and National Insurance contributions), or by a proxy (generally a family member). While many employ a PA using Local Authority (LA) funding in the form of a DP, others pay for a PA privately using their own resources.

In 2020/2021, approximately 70,000 people (30% of all people receiving DPs) were eligible for LA funding to help with achieving well‐being goals (after meeting needs and means‐tested criteria) by directly employing their own care worker(s) (Skills for Care, 2021a). Employers of PAs are subject to the same employer rights and responsibilities as others, although few undertake employer‐related training (Skills for Care, 2021b), despite the administration involved (McGuigan et al., 2016). In response to early calls for user‐led or peer‐support organisations (see Evans, 1992), disability support organisations, including Centres for Independent Living (CILs) and brokerage agencies, were funded by some LAs to support PA employers, or assist with support planning (Williams et al., 2014). Such support may be user‐led, covering assistance with recruiting PAs, providing training, managing DPs and administrative responsibilities such as signposting to payroll services. In a survey of 1636 individual employers, almost half of respondents (46%) had accessed information from a disability or local support organisation in 2020/2021 (Skills for Care, 2021b); although this may reflect the distribution of its survey via support organisations. Currie et al. (2016) reported LAs finding it difficult to commission such personalised provider organisations (i.e. peer‐led brokerage services) due to funding cuts and closures. While valued, such peer‐support organisations are in decline in the UK (Hyslop et al., 2020).

Covid‐19 created multiple challenges for people with care and support needs who faced increased risks of physical and mental health deterioration, severe illness, hospitalisation and death (Comas‐Herrera et al., 2020). To date, much attention has focussed on care home residents or specific user groups such as people affected by dementia and carers (Giebel et al., 2021). PA employers' experiences are under‐researched, and little is known about their care and support needs during the pandemic, or the sources of support that were available to them during this time. Disabled people felt largely forgotten during the pandemic at global and national levels (Courtenay & Perera, 2020; Shakespeare et al., 2021; Shakespeare et al., 2022). A study of PAs in the early months of Covid‐19 in England found that many PAs decided to stop working or were asked to stop working by their employer due to the risk of virus transmission (Woolham et al., 2020). Other PAs, in contrast, were sometimes replaced by a family member because care needs still had to be met. Of those retained by their employer, PAs reported very limited work‐related information or support during the early months of the pandemic, such as information about the virus, practical arrangements including getting tested for Covid‐19 or accessing personal protective equipment (PPE), and some reported lack of clarity about their ‘keyworker status’ (ibid, 2020), categorised as those working in ‘essential’ occupations and industries (i.e. 31% of key workers worked in health and social care) (Office for National Statistics, 2021).

Our objective in this study was to understand the views and experiences of people working in disability support organisations that provide support to people employing PAs and to learn if and how these organisations adapted in response to the needs of disabled people employing social care PAs during the Covid‐19 pandemic. We sought to provide a preliminary overview of the questions that participants working in these organisations found were frequently raised by PA employers, and also how the work of support organisations at local level was changing in response to the pandemic. This study was a preliminary to conducting a wider ongoing study of PA employers and informed data collection with individual employers; these findings are reported elsewhere (Leverton, Samsi, Woolham, & Manthorpe, under review).

## 2 METHODS

### 2.1 Design and recruitment

This qualitative study was part of a wider 17‐month study (reported in Leverton, Samsi, Woolham, & Manthorpe, under review). In this phase, semi‐structured interviews were conducted with staff working in organisations that provide support to people employing PAs, including user‐led disability support organisations, brokerage agencies and LA‐linked support services (i.e. payroll services). We used a purposive sampling technique, initially identifying support organisations that varied by geographical location, type of organisation (i.e. CILs; brokerage agencies; or LA‐linked teams), type of support provided (i.e. DP support; help with recruiting PAs). We also contacted support organisations by phone or email that had taken part in a previous study of PAs in 2018 (Woolham et al., 2019), who had given permission to be contacted again for further research. We provided information on the study by email, including contact information for the research team for potential participants to express interest, and asked for our study information to be shared with other staff. We purposively sought staff in different roles to gain a range of perspectives. Potential participants were offered the choice to be interviewed by telephone or video call via Zoom conference software. Minimal risk University ethics was sought and approved by King's College London [Ref: MRA‐20/21‐21647].

### 2.2 Data collection

Interviews took place between March and July 2021. Participants had the opportunity to ask questions about the study, before providing informed consent. Participants were assured that their data would be handled securely, and informed of their right to withdraw from the study. The consent process was audio‐recorded and stored separately to the interview recording to uphold confidentiality and anonymity. Participants were asked to verbally respond to a short demographics form before beginning the interview. A semi‐structured interview guide was used to cover main topic areas, including: (1) an overview of their role; (2) implications of Covid‐19 on their work; (3) contact with LAs and other support sources; (4) payment for PAs; (5) advice and support for PA employers; (6) future considerations. Interviews lasted up to one‐hour and were audio‐recorded (with consent) and transcribed verbatim. Participants were thanked for their time and posted/emailed a £20 shopping voucher.

### 2.3 Data analysis

Interview transcripts were checked and pseudo‐anonymised to protect the privacy of participants and their workplaces. All transcripts were then read in detail and Framework Analysis (Ritchie & Spencer, 1994) was applied to look for patterns and themes across the data. This approach is extensively applied in social and health sciences research as a robust method used to capture the experiences of participants (Parkinson et al., 2016). Leverton conducted the primary data analysis, with secondary support from Samsi, following the five‐step approach to Framework Analysis: familiarisation; identifying a thematic framework; indexing; charting; and interpretation (Ritchie & Spencer, 1994). Full team discussions were held to check and validate the initial framework and developing themes. The research team held varied backgrounds and perspectives, including social care researchers with experience in DP studies, homecare and workforce research; minimising the risk of interpretation bias. The advisory group included people using DPs, social care support organisations and social work practice.

## 3 FINDINGS

### 3.1 Participant demographics and organisation information

We interviewed 15 participants, working across 11 organisations in England, who provide support to PA employers, amongst other activities. Staff roles included Chief Executive Officers, a Chief Operating Officer, DP Team Leaders/Workers, Personal Budget Coordinator, Managers, a Senior Commissioning Manager, a Director, Administrators and a Senior Information Officer. Six participants employed their own social care PA. All but one of the 11 organisations were user‐led registered charities, such as CILs or disability support organisations; the remaining organisation was an LA‐linked DP service. Organisations covered geographically varied regions of England. Table 1 provides participant demographic and organisation information.

**TABLE 1:** Participant demographic and organisation information

	Participants (*N* = 15)
Gender	
Male	5
Female	10
Age	
30–40	3
41–50	7
51–60	2
60–75	3
Ethnicity	
White–British/English/Welsh/Scottish/Northern Irish	12
White–Irish	2
Asian/Asian British–Indian	1
Organisation location	
London	3
South East	3
East of England	1
West Midlands	1
Yorkshire and the Humber	2
North West	4
North East	1
Duration in current role^a^	
1–3 years	3
3–5 years	1
5–10 years	2
10 years+	7

^a^ Two participants did not report duration in current role.

### 3.2 Themes

In response to our study objectives, we identified one overarching theme demonstrating the impact of Covid‐19 on the work of disability support organisations, titled ‘Working to prevent and challenge marginalisation of PA employers’. Across three related subthemes, we describe how participants worked to achieve this by (1) Advocating for the voice of a ‘forgotten’ group; (2) Needing to be proactive and (3) Adapting to new tasks and ways of working. Each subtheme is summarised in Table 2 and detailed after with supporting quotes for context.

**TABLE 2:** Summary of subthemes and coding framework

Subthemes	Coding framework
(1) Advocating for the voice of a ‘forgotten’ group	
(2) Needing to be proactive	(a) Advocating for the people, by the people (b) Sensemaking and filtering misinformation (c) Supporting PAs with the direct payments (DPs) system
(3) Adapting to new tasks and ways of working	(a) Creative solutions to overcome barriers to supporting PA employers (b) Reflecting on the longevity of remote working

## 4 WORKING TO PREVENT AND CHALLENGE MARGINALISATION OF PA EMPLOYERS

Participant accounts described ways in which people with care and support needs were disadvantaged throughout the pandemic. Whilst many of these reported problems existed long before Covid‐19, they were perceived to have been heightened and exacerbated during this time. New and changed ways of working were discussed in relation to how these organisations responded to the needs of PA employers.

### 4.1 Advocating for the voice of a ‘forgotten’ group

PA employers were perceived as a largely forgotten group, particularly in the early months of the pandemic. Recognising that PAs and their employers were left out of early decisions and guidance, participants tried to represent the voices of this group at local and national levels. Participants said they had actively campaigned and worked closely with their LA adult services department to advocate for the inclusion of PA employers and the PA workforce in ongoing arrangements and crisis planning throughout the pandemic:

“Behind the scenes, what we were doing was constantly campaigning to social services to treat PA employers equally and carry that knowledge that these keyworkers [PAs] needed as much protection as people working in any other part of the care sector, including their own.” [Participant 14]

Crisis planning and initial arrangements focused around people living in care homes or those in hospitals, while people receiving care in their own home were perceived as an ‘after thought’. Participants believed this was evidenced by the exclusion of PAs in the initial instructions from central government and LAs when talking of ‘essential’ or ‘key workers’. The consequences of this were reported by participants whose work involved supporting PA employers to navigate guidance around social distancing, while needing continued support from their PA. For example, without PAs being recognised as essential workers early on, employers faced the risk of their PAs being unable to access priority shopping hours during the first national lockdown (and so were having to mix with more people at busier times), and had found it difficult to acquire Personal Protective Equipment (PPE) or testing for Coronavirus for themselves and their PAs. When reflecting on conversations with PA employers, particularly in the first few months of the pandemic, participants described high levels of stress resulting from government guidance, with many feeling unable to manage without their PA:

“They weren't sure what they should do with their PAs, whether they could tell them not to come for work, but then how would they get their support?” [Participant 11]

Participants indicated that stress could also be the consequence of insufficient, delayed or unclear information/guidance relevant to those employing PAs. Frustrations in general turned towards the perceived lack of acknowledgement of the PA workforce and understanding of the needs of PA employers by central and local government:

“When the pandemic started, I think the general impression was that the Government wasn't really acknowledging the impact on disabled people generally, but in particular, I think PA employers were sort of way down the list of people who were given any consideration at all really… it's something that gets a bit forgotten anyway.” [Participant 07]

Many participants felt that closer working between central/local government and disability support organisations from the start (and pre‐dating Covid‐19) would have ensured that the needs of PA employers were recognised:

“If the central Government had just had a conversation with organisations like us from the get‐go…. I definitely believe that that is the solution, rather than it being firefighting and reactive, it could have been proactive.” [Participant 04]

Furthering this, none of those interviewed referred to being involved in the many arrangements for civil contingencies that had existed prior to Covid‐19, such as local resilience forums or being involved in emergency planning. Likewise, none referred to any of their own organisation's possible contingency planning for disasters or similar events.

### 4.2 Needing to be proactive

To respond to the challenges facing PA employers throughout the pandemic, participants mentioned taking a proactive approach whereby support organisations attempted to anticipate and quickly react to pressing issues arising from Covid‐19. This included problems around information needs, such as making sense of large quantities of information from central government and LAs, filtering misinformation on social media, and supporting PA employers to navigate the DP system in an unprecedented and chaotic time.

#### 4.2.1 Advocating for the people, by the people

Many participants identified as a disabled person, with six employing their own PAs. As such, they described being able to anticipate the needs of people employing PAs at the start of the pandemic and throughout, and acting quickly to provide support and advice:

“…we know that all these things matter because we have disability and long‐term health conditions too. So 61% of our workforce have got lived experience of disability or a long‐term health condition, and many of us also employ our own PAs.” [Participant 04]

These anticipated support and information needs included accessing food and medication (particularly when many PA employers were shielding or remained indoors), information on mental health support, accessing PPE and testing. One participant reported that their colleagues worked closely with the local community to make sure information was wide‐reaching:

“A number of our staff became local COVID Community Champions, so that they were able to share information around PPE and the vaccine, so again that is to sort of demonstrate that local presence and that local knowledge that we can share.” [Participant 03]

The need to be proactive was reported as a response to a lack of timely guidance and information coming from central government and LAs. As noted above, many contrasted the focus on care homes and the NHS to the lack of focus on disabled people living at home.

Working relationships with LAs varied. Some participants spoke of a close working relationship between their organisation and the LA that had existed before the pandemic and continued throughout. In contrast, others felt disappointed with the lack of collaboration between their organisations and LAs. While acknowledging that lack of interference or oversight may have been valued by PA employers pre‐Covid, many participants thought the government and LAs were disconnected during the pandemic and too slow in responding to the needs of PA employers. Some participants described feeling much of their work was substituting for what they perceived LAs should have been doing:

“In terms of supporting people during a pandemic, it was about, ‘I don't understand the process of it, we've not been told anything about… there's been a misinformation, I've not heard from a LA at all’. So at the beginning of the pandemic, there was that initial consultation with people… we set up a hotline saying, ‘What support do you need?’.” [Participant 02]

With high numbers of disabled people working in the organisations we spoke to, participants voiced concerns for the mental health and wellbeing of their colleagues who were shielding or isolating. They acknowledged this was also a challenge for PA employers; many of whom were unable to leave their homes or meet support networks, including their PAs. Several participants reported that their organisation had set up befriending and/or peer‐support services in response to loneliness and expressed hope for these services to continue beyond the pandemic, despite uncertainty of funding sources.

#### 4.2.2 Sensemaking and filtering misinformation

Most participants confirmed that LAs did provide information for PA employers during the pandemic, but not always expediently or clearly. They agreed with many PA employers who described this information as too long, overcomplicated or not specific to the needs of individual employers and their employment responsibilities:

“I think we find [LA] will sometimes send out quite technically worded letters. So, the original thing was ‘if you want your PAs to be vaccinated, here's a really long email explaining the process in small print and then here's – we've attached a spreadsheet that you can fill in with all these details’.” [Participant 07]

Consequently, participants described working quickly to filter out salient points from information provided by government and LAs to help keep PA employers updated. Many organisations developed accessible resources, including newsletters and emails with up‐to‐date information, ‘Covid‐19 Frequently Asked Questions’ webpages, and online and community ‘hubs’; greatly increasing participants' workload:

“It was an incredibly busy six months in particular; I had one weekend off in about six months.” [Participant 12]

Participants also described battling with social media information, reporting this as the main source of misinformation reaching PA employers. Some organisations ensured an active presence on social media during the pandemic, trying to correct misinformation and provide up‐to‐date guidance to employers beyond those who were known to, or knew of, their organisation. Access to Covid‐19 testing was described by one participant as such a topic:

“Covid tests was a common misconception, they [PA employers] were told that they had to pay for a private test. They'd got that from social media, and a lot of disabled people do rely on social media as a source of their information, but there was a lot of misinformation.” [Participant 02]

#### 4.2.3 Supporting PA employers with the direct payments (DPs) system

Participants reported receiving many questions about DPs from PA employers throughout the pandemic. These questions concerned payment for PAs who stopped working during national lockdowns; whether the employer should continue paying PAs either their full wage or sick pay when needing to self‐isolate; ability to use the government's furlough scheme (paying workers who were not working); paying for PPE from their DP; how, and whether employers could use any surplus that had built up from not paying their PA for any unworked time.

All participants agreed with PA employers about a lack of clarity during the pandemic regarding appropriate use of DPs. Some empathised with frustrations expressed by PA employers regarding the DP system, acknowledging that while rules or conditions existed pre‐pandemic, they continued at a time when flexibility might have eased many of the concerns that PA employers faced throughout the pandemic, particularly during times of national lockdown and tightened restrictions:

“I think that when there's a major national emergency, a pandemic, when especially disabled and older people are panicking and are very much having to deal with an awful lot, the LA needs to be more flexible.” [Participant 06]

When guidance on paying PAs was not readily available from central government earlier on in the pandemic, participants reported that some support organisations and LAs decided to use personal discretion when national guidance seemed inappropriate. This included advising employers to continue paying their PA even if they had stopped work. One participant reported “having to just do things that we thought were right”. With known difficulties around recruiting and retaining PAs, participants felt it was important that employers should retain their PAs for a time when both the employer and PA felt it was safe to return to work. In some areas, this view was shared by their LA:

“I think I would applaud the LA locally… the position the LA came out in September [2020] and said that they didn't want any PA to be financially disadvantaged for any Covid‐related absence if they were required to self‐isolate for up to a 14‐day period, and that they would be paid full pay for that, and not have to turn to statutory sick pay.” [Participant 1]

One participant from an LA‐linked DP team described how emergency funding was made available to PA employers to ensure they could retain their PA. This was applicable when PAs were required to shield or felt unsafe working, but their employer was unable to manage without support and turned to more expensive homecare agency staff, or paid a relative to provide care, whilst continuing to pay their PA.

However, this was not standard across all LAs, nor did such information always reach PA employers. Some participants voiced their frustration with the LA, perceiving there was a lack of clarity and inconsistent information for PA employers. One common example given by participants concerned resourcing of PPE, with many PA employers reportedly paying for PPE from their own pockets, while others were told by their LA/DP advisor to use their DP monies. This lack of clear and timely information related to the surprisingly ‘quiet’ early months of the pandemic, when fewer PA employers than expected had been in touch with participants' organisations for advice or support. Several participants deciphered undertones of PA employers being fearful of being caught out for unknowingly doing the wrong thing and having their DPs stopped or clawed back by the LA as a result:

“There's a lot of scaremongering which we've picked on ‐ ‘If we dare speak out, our funding will stop,’ is a consistent remark we get. If a disabled person speaks out… they've got it into their heads that the Council [LA] will take their money away from them.” [Participant 02]

This further fed into the actions of some support organisations that worked quickly to get accurate and accessible information out to PA employers, as well as holding a greater public presence to dispel myths and ease concerns around DP use. Participants reported minimal contact with employers self‐funding their PA support, and perceiving there to have been very little, if any, guidance or support for these employers during the pandemic. Many felt that self‐funders were rarely on the radar of support organisations or LAs, despite the LAs' obligations to provide them with advice and information.

### 4.3 Adapting to new tasks and ways of working

Covid‐19 abruptly changed not only the everyday tasks of participants but also the way that participants worked. Whilst working remotely created various challenges to supporting PA employers that participants tried to overcome, the ability to work from home was described as a welcomed change, particularly by participants who identified as disabled people.

#### 4.3.1 Creative solutions to overcome barriers to supporting PA employers

While some new tasks such as wellbeing checks and liaising with insurance companies became more frequent, participants reported that much of their normal work before the pandemic had substantially reduced or became more difficult to achieve remotely, such as supporting people to recruit PAs and visiting them at home to help them complete documentation. Approaches taken by support organisations to overcome these issues were described; for some this included changes such as an easing of previous data protection rules so that staff in these organisations could share the names of PAs and employers with whom they were in contact with the LA. Others began moving towards more systematic practices, including collecting names of PAs to form a register or list (something not done previously), so that people working as PAs could access to the same resources as other essential or key workers, including PPE, testing and early access to the vaccine which participants felt was a needed change, given the situation:

“We were actually able to supply our LA partners and CCGs [NHS Clinical Commissioning Groups] with a list of individual employers, and who the PAs were and their address and contact details, which enabled LAs to directly write out to PAs, to let them know what the procedure was.” [Participant 03]

Moreover, while digital working became the new normal for many workers in the UK, participants described a ‘digital‐divide’ as newly important during the pandemic. Some viewed the shift to remote working and online communications as further marginalising of an already vulnerable group of people, many of whom were not able to use or access technology or the internet:

“A lot of people have not had the information. Right at the beginning information was just sent out to people who had email addresses, so there were a significant number of people who don't have email addresses or who aren't able to access email on a regular basis.” [Participant 05]

A small number of participants remarked that digital working was also more challenging if people needed translators or interpreters and/or lacked online access. This applied to completing documents to set up or manage their DPs:

“In the past it was a lot easier to explain things to people face‐to‐face. That's gone now and I think a lot of those people are probably not as well informed as they would have been otherwise.” [Participant 06]

This divide made it even more important for support organisations to work quickly and proactively in sharing information widely and maintaining a local presence within communities, as described above.

#### 4.3.2 Reflecting on the longevity of remote working

Despite these challenges, many participants spoke about the positive impact that Covid‐19 had on their own work life, including the view that working remotely and digitally was a welcome change that often helped to speed up tasks and improved accessibility for disabled staff, some of whom did not previously have this as an option:

“…it's given us a lot more flexibility to do that. I mean, I've got to be honest, personally, it was getting hard getting out of the car every day and, yeah, it's winter, I've got to drag my wheelchair and do all that kind of stuff. Being able to work from home two or three days a week has been very beneficial.” [Participant 13]

However, for some, this was overshadowed by concerns that prolonged or continued remote working would heighten isolation and take its toll on mental wellbeing:

“It was tough, like, the mental side of not seeing anybody, so you had to learn your own coping strategies as well as working and supporting other people.” [Participant 8]

## 5 DISCUSSION

### 5.1 Summary of main findings and implications for policy and practice

Through qualitative interviews, we sought to understand the views, experiences and impact on the role of people working in organisations supporting PA employers during the Covid‐19 pandemic. Much of the work carried out by disability support organisations throughout the pandemic focused on preventing and challenging the marginalisation of an already disadvantaged and vulnerable group. Participants reported working proactively from the start of the first national lockdown, anticipating the needs of PA employers and delivering information in a more person‐centred and timely way than they felt was being achieved by central and local government. Similarly, reporting on the experiences of disabled people using social care in the time of Covid‐19, Pearson et al. (2022) suggested that the failings of LAs were detrimental to independent living for people with care and support needs. It was a common perception that participants in our study felt their work was compensating for the failings of LAs.

While it was generally agreed that LAs did provide information for PA employers during the pandemic, this was judged as overcomplicated, too slow and not specific to the needs of PA employers. This extended to a view that those working in LAs and government did not understand the needs of disabled people, nor the work carried out by PAs, warranting ongoing communications and closer working between disability support organisations and central and local government. In line with this, Pearson et al. (2022) reported that a lack of understanding around the PA workforce, the ILM and the DP system was evidenced by the insufficient responses of many LAs towards disabled people's support needs throughout the pandemic.

Participants discussed often using personal judgement when advising employers of what they perceived to be the best thing to do in the absence of appropriate formal guidance; namely, helping employers to maintain a relationship with their PA throughout the pandemic, which required to be continued payment even if the PA had stopped working (i.e. in times of national lockdown). The International Federation of Social Workers echoed this as an approach taken by social workers when deciding whether to follow official guidance and organisational policies during Covid‐19 (Banks et al., 2020). This points strongly to the absence of these workers and their voices in the development of Covid‐19 policies and practice decisions. In light of this, participants viewed a key part of their role as advocating for PA employers, by campaigning and positioning themselves in meetings with central/local government to ensure the voices of those with care and support needs were reflected in Covid‐19 planning and arrangements.

Participants reported holding a greater presence across social media platforms to dispel misinformation, and within local communities, setting up advice hubs and befriending services. The use of social media as a source of support for many PA employers throughout the pandemic suggests not only a lack of more formal support, but points towards a desire for peer‐support amongst some PA employers. A recent report highlighted peer‐support as one of 12 positive influences on the uptake of DPs (TLAP, 2022). For people with DPs, peer‐support networks and organisations (e.g. user‐led groups) are reported to enhance confidence and communication, foster collaboration and draw on specialist knowledge from those with lived experience. Whilst such support is often the premise behind user‐led organisations, how to sustain and help such organisations to flourish is not yet understood (TLAP, 2022).

The onset of Covid‐19 saw a change in the tasks participants carried out within their organisation to assist PA employers. While tasks such as befriending and wellbeing services became more frequent, other tasks including supporting employers to recruit PAs became more challenging during the pandemic, particularly if there were language or digital barriers. Covid‐19 brought to light the importance of social media and online connectedness (Shakespeare et al., 2022), yet participants perceived that many people with care and support needs, including PA employers, missed important information during the pandemic if they did not have access to the internet. Disabled people globally were found to be particularly disadvantaged by the digital‐divide caused by remote working during Covid‐19, especially in the first wave of the pandemic (Jesus et al., 2021; Mehrotra, 2021). ‘Good’ disability support organisations were reported as those who not only anticipated the threat of a digital‐divide, but worked quickly to minimise it, that is by reallocating budget to fund online access and electronic notebooks for people at risk (Shakespeare et al., 2022). Participants reported creative solutions taken by their support organisations to minimise this divide, such as holding a physical presence within local communities (e.g. Covid‐19 hubs) to reach wider audiences.

### 5.2 What does this mean in the current context?

It is important to consider the context in which these findings are embedded. The DP system, with its rejection of ‘services’ and transfer of power to enable service users to employ staff of their choice and under their direction, emerged directly from the ILM. Prior to the pandemic, lack of interference or regulation was what many PA employers seemed to value to sustain choice and control (Woolham et al., 2019). When the pandemic began, it was seemingly difficult for LAs and the NHS to identify individual employers and PAs. In this study, we heard that some support organisations provided LAs with names and address of PAs and their employers to keep them connected and informed. This is in contrast with the initial premise of the ILM and the notion of choice and control afforded to DP users. However, learning from the challenges posed by Covid‐19, some level of regulation or registration may be warranted (albeit with close monitoring and evaluation) (Manthorpe et al., 2021), particularly when situations arise where individual employers and directly employed workers would benefit from greater connectedness with the LAs that are funding this transaction.

While this study suggests that from the point of view of support organisations many PA employers were ‘forgotten’ or abandoned by LAs, other studies have indicated that contact with health and social care professionals, such as social workers, was maintained in some ways, for example by ‘welfare calls’ or telephone contact (Tuijt et al., 2021). This suggests the picture of isolation may be incomplete, or those DP users were seen as able to manage independently; research is presently exploring their perspectives (Leverton, Samsi, Woolham, & Manthorpe, under review). Moreover, although participants reported a lack of collaboration between central/local government and disability support organisations in crisis planning, these organisations' own contingency planning was not evident, highlighting a nationwide lack of preparedness to plan and develop useful protocols, policies and guidance that are inclusive of disabled people in times of crisis (Boin et al., 2020). These failings provide important lessons for systemic and organisational change to ensure effective contingency planning that is inclusive of people with care and support needs, including those living in the community.

From the outset of the ILM, support organisations were pinpointed as key to successful policy implementation (Department of Health, 2000; Evans & Hasler, 1996), yet organisations such as CILs have struggled to secure sufficient funding to expand and reach diverse user populations (Pearson, 2004). More recent reports have shown that LAs struggle to commission personalised provider organisations (e.g. peer‐led brokerage services) due to widescale funding cuts (Currie et al., 2016). The need for their own LA‐linked services to provide support to people employing PAs was argued by participants. They seemed to be developing a case for sustained funding for both advocacy activities and services for PA employers.

One gap in the narrative was the position of the many people privately funding their PAs. They remain largely unknown to LAs but also to organisations that might be able to support them. While participants described a clear commitment to PA employers and disabled people more widely, this did not extend to PAs who were not regarded as within the remit of the organisations interviewed, other than the LA‐linked service.

### 5.3 Strengths and limitations

This is, to our knowledge, the only study to explore the impact of the work carried out by staff working in organisations that supported PA employers during the Covid‐19 pandemic. We included organisations from geographically diverse locations across England, and interviewed participants working in varied roles to ensure a representative sample. However, there are limitations to this study. First is the risk of this being a self‐selecting participant group with self‐reported data. Second, there are limitations relating to recruitment, including an absence of staff working in support organisations offering brokerage services, as well as a lack of applicability to employers privately funding their PAs. This potentially reflects an under‐representation of these services in England, and a sparsity of support for individuals self‐funding their PAs. Third, is the lack of PA employer voices to reflect on the support they received from these organisations (reported separately elsewhere), and the lack of LA perspectives on their own pandemic actions for people using DPs to fund their care.

## 6 CONCLUSION

Prior to Covid‐19, the complications of managing DPs were observed in many studies of PA employers. This present paper provides another perspective from staff working in organisations seeking to offer support to PA employers during the pandemic in England. We further highlighted the frustrations experienced by staff in these organisations around the perceived inflexibility of the DP system, as well as the proactive approaches (e.g. befriending services) they used to support some employers through this challenging time. While a small‐scale study, it has implications for local and central government in considering ways of collaborative working with disability support organisations to better understand and respond to the needs of disabled people. Our findings suggest systemic and organisational change is required to embed effective contingency planning into policies and guidance for planning, with and for people with care and support needs residing in the community. The data reported here suggest areas where such planning might usefully be focussed and the potential for specific services or organisations to be commissioned to provide such support.

## AUTHOR CONTRIBUTIONS

KS was Principal Investigator; KS, JW and JM contributed to the study design. ML was responsible for conducting the study, including data collection and analysis. KS contributed to the development of themes, and JW and JM provided support with interpreting the findings. ML led the writing of the manuscript, and all authors contributed to revisions in producing the final manuscript.

## FUNDING INFORMATION

This research was funded by the National Institute for Health and Care Research (NIHR) School for Social Care Research (SSCR). This study is supported by the National Institute for Health and Care Research (NIHR) Applied Research Collaboration South London (NIHR ARC South London) at King's College Hospital NHS Foundation Trust. The views expressed are those of the author(s) and not necessarily those of the NIHR or the Department of Health and Social Care.

## CONFLICT OF INTEREST

The authors declare no conflict of interest.

## Data Availability

Research data are not shared.

## References

[hsc14098-bib-0001] Banks, S. , Cai, T. , de Jonge, E. , Shears, J. , Shum, M. , Sobočan, A. M. , Strom, K. , Truell, R. , Úriz, M. J. , & Weinberg, M. (2020). Ethical challenges for social workers during Covid‐19: A global perspective. International Federation of Social Workers. https://www.ifsw.org/wp‐content/uploads/2020/07/2020‐06‐30‐Ethical‐Challenges‐Covid19‐FINAL.pdf

[hsc14098-bib-0002] Boin, A. , Lodge, M. , & Luesink, M. (2020). Learning from the COVID‐19 crisis: An initial analysis of national responses. Policy Design and Practice, 3(3), 189–204. 10.1080/25741292.2020.1823670

[hsc14098-bib-0003] Comas‐Herrera, A. , Fernandez, J. L. , Hancock, R. , Hatton, C. , Knapp, M. , McDaid, D. , Malley, J. , Wistow, G. , & Wittenberg, R. (2020). COVID‐19: Implications for the support of people with social care needs in England. Journal of Aging & Social Policy, 32(4–5), 365–372. 10.1080/08959420.2020.1759759 32497462

[hsc14098-bib-0004] Courtenay, K. , & Perera, B. (2020). COVID‐19 and people with intellectual disability: Impacts of a pandemic. Irish Journal of Psychological Medicine, 37(3), 231–236. 10.1017/ipm.2020.45 32404232 PMC7287305

[hsc14098-bib-0005] Currie, R. , Kinn, A. , Thompson, R. , & Hamilton, S. (2016). An evaluation of my support broker final report. McPin Foundation. https://media.nesta.org.uk/documents/msb_final_report.pdf

[hsc14098-bib-0006] Department of Health . (2000). Community care (direct payments) act 2996: Policy and practice guidance (2nd ed.). Department of Health.

[hsc14098-bib-0007] Evans, J. (1992). The role of Centres of independent/integrated living and networks of disabled people. In C. Barnes (Ed.), Making our own choices, independent living, personal assistance and disabled people (pp. 59–63). British Council of Disabled People. https://disability‐studies.leeds.ac.uk/wp‐content/uploads/sites/40/library/Barnes‐making‐our‐own‐choices.pdf

[hsc14098-bib-0008] Evans, J. , & Hasler, F. (1996). Direct payments campaign in the UK. *Presentation for the European Network on Independent Living Seminar*. Stockholm: 9–11 June.

[hsc14098-bib-0009] Fleming, P. , McGilloway, S. , Hernon, M. , Furlong, M. , O'Doherty, S. , Keogh, F. , & Stainton, T. (2019). Individualized funding interventions to improve health and social care outcomes for people with a disability: A mixed‐methods systematic review. Campbell Systematic Reviews, 15(1–2), 1–77. 10.4073/csr.2019.3 PMC8356501 37131462

[hsc14098-bib-0010] Giebel, C. , Lord, K. , Cooper, C. , Shenton, J. , Cannon, J. , Pulford, D. , Shaw, L. , Gaughan, A. , Tetlow, H. , Butchard, S. , & Gabbay, M. (2021). A UK survey of COVID‐19 related social support closures and their effects on older people, people with dementia, and carers. International Journal of Geriatric Psychiatry, 36(3), 393–402. 10.1002/gps.5434 32946619 PMC7536967

[hsc14098-bib-0011] Hyslop, J. , Aveyard, H. , Abreu, G. , & Appleton, J. V. (2020). How do peer networks support people with personal budgets? A review of the research evidence from the UK. Disability and Society, 35(1), 25–51. 10.1080/09687599.2019.1601069

[hsc14098-bib-0012] Jesus, T. S. , Bhattacharjya, S. , Papadimitriou, C. , Bogdanova, Y. , Bentley, J. , Arango‐Lasprilla, J. C. , & Kamalakannan, S. (2021). Lockdown‐related disparities experienced by people with disabilities during the first wave of the COVID‐19 pandemic: Scoping review with thematic analysis. International Journal of Environmental Research and Public Health, 18(12), 6178. 10.3390/ijerph18126178 34200979 PMC8228347

[hsc14098-bib-0013] Leverton, M. , Samsi, K. , Woolham, J. , & Manthorpe, J. (under review). “I have enough pressure as it is, without the worry of doing something wrong because of ignorance”. The impact of Covid‐19 on people who employ social care Personal Assistants.

[hsc14098-bib-0014] Manthorpe, J. , Samsi, K. , Norrie, C. , & Woolham, J. (2021). Caring in Covid‐19: Personal Assistants' changing relationships with their Clients' family members. Journal of Long‐Term Care, 256–263. 10.31389/jltc.77

[hsc14098-bib-0015] McGuigan, K. , McDermott, L. , Magowan, C. , McCorkell, G. , Witherow, A. , & Coates, V. (2016). The impact of direct payments on service users requiring care and support at home. Practice: Social Work in Action, 28(1), 37–54. 10.1080/09503153.2015.1039973

[hsc14098-bib-0016] Mehrotra, N. (2021). Emergent disability voices on social media during COVID‐19 times. Disability and the Global South, 8(1), 1993–2006.

[hsc14098-bib-0017] Miller, R. , Glasby, J. , & Dickinson, H. (2021). Integrated health and social Care in England: Ten years on. International Journal of Integrated Care, 21(4), 6. 10.5334/ijic.5666 PMC8555479 34754282

[hsc14098-bib-0018] Office for National Statistics . (2021). Coronavirus and key workers in the UK. Data and analysis from Census 2021. https://www.ons.gov.uk/employmentandlabourmarket/peopleinwork/earningsandworkinghours/articles/coronavirusandkeyworkersintheuk/2020‐05‐15

[hsc14098-bib-0019] Parkinson, S. , Eatough, V. , Holmes, J. , Stapley, E. , & Midgley, N. (2016). Framework analysis: A worked example of a study exploring young people's experiences of depression. Qualitative Research in Psychology, 13(2), 109–129. 10.1080/14780887.2015.1119228

[hsc14098-bib-0020] Pearson, C. (2004). The implementation of direct payments: Issues for user‐led organisations in Scotland. In C. Barnes & G. Mercer (Eds.), Disability policy and practice: Applying the social model. Disability Press.

[hsc14098-bib-0021] Pearson, C. , Watson, N. , Brunner, R. , Cullingworth, J. , Hameed, S. , Scherer, N. , & Shakespeare, T. (2022). Covid‐19 and the crisis in social care: Exploring the experiences of disabled people in the pandemic. Social Policy and Society, 1–16. 10.1017/S1474746422000112 PMC8446989 34548712

[hsc14098-bib-0022] Ritchie, J. , & Spencer, L. (1994). Qualitative data analysis for applied policy research. In B. Bryman & R. Burgess (Eds.), (pp. 173–194). Analyzing qualitative data.

[hsc14098-bib-0023] Shakespeare, T. , Ndagire, F. , & Seketi, Q. E. (2021). Triple jeopardy: Disabled people and the COVID‐19 pandemic. The Lancet, 397(10282), 1331–1333. 10.1016/S0140-6736(21)00625-5 PMC7963443 33740474

[hsc14098-bib-0025] Shakespeare, T. , Watson, N. , Brunner, R. , Cullingworth, J. , Hameed, S. , Scherer, N. , Pearson, C. , & Reichenberger, V. (2022). Disabled people in Britain and the impact of the COVID‐19 pandemic. Social Policy & Administration, 56(1), 103–117. 10.1111/spol.12758 34548712 PMC8446989

[hsc14098-bib-0026] Skills for Care . (2021a). The state of the adult social care sector and workforce in England. Skills for Care. https://www.skillsforcare.org.uk/adult‐social‐care‐workforce‐data/Workforce‐intelligence/documents/State‐of‐the‐adult‐social‐care‐sector/The‐State‐of‐the‐Adult‐Social‐Care‐Sector‐and‐Workforce‐2021.pdf

[hsc14098-bib-0027] Skills for Care . (2021b). Individual employers and the personal assistant workforce. Skills for Care. https://www.skillsforcare.org.uk/adult‐social‐care‐workforce‐data/Workforce‐intelligence/documents/Individual‐employers‐and‐the‐PA‐workforce/Individual‐employers‐and‐the‐PA‐workforce.pdf

[hsc14098-bib-0028] TLAP . (2022). Better direct payments: From insight to action. Social Care Institute for Excellence. https://www.thinklocalactpersonal.org.uk/_assets/Reports/Better‐Direct‐Payments.pdf

[hsc14098-bib-0029] Tuijt, R. , Frost, R. , Wilcock, J. , Robinson, L. , Manthorpe, J. , Rait, G. , & Walters, K. (2021). Life under lockdown and social restrictions‐the experiences of people living with dementia and their carers during the COVID‐19 pandemic in England. BMC Geriatrics, 21(1), 1–12. 10.1186/s12877-021-02257-z 33971847 PMC8107803

[hsc14098-bib-0031] Williams, V. , Porter, S. , & Marriott, A. (2014). Your life, your choice: Support planning led by disabled people's organisations. British Journal of Social Work, 44(5), 1197–1215. 10.1093/bjsw/bct005

[hsc14098-bib-0032] Woolham, J. , Samsi, K. , Norrie, C. , & Manthorpe, J. (2020). The impact of the coronavirus (Covid‐19) on people who work as social care pPersonal aAssistants. In Health and social care workforce research unit, the policy institute, King's College London. NIHR Policy Research.

[hsc14098-bib-0033] Woolham, J. G. , Norrie, C. M. , Samsi, K. , & Manthorpe, J. (2019). Roles, responsibilities, and relationships: Hearing the voices of personal assistants and directly employed care workers. Health and social care workforce research unit, the policy institute, King's College London. NIHR Policy Research.

